# The role of bacterial infections in rheumatoid arthritis development and novel therapeutic interventions: Focus on oral infections

**DOI:** 10.1002/jcla.24897

**Published:** 2023-05-24

**Authors:** Shima Afrasiabi, Nasim Chiniforush, Alireza Partoazar, Ramin Goudarzi

**Affiliations:** ^1^ Laser Research Center of Dentistry Dentistry Research Institute, Tehran University of Medical Sciences Tehran Iran; ^2^ Experimental Medicine Research Center Tehran University of Medical Sciences Tehran Iran; ^3^ Division of Research and Development, Pharmin USA, LLC San Jose California USA

**Keywords:** anti‐citrullinated protein antibodies, inflammation, microbiota, immune dysregulation, infections, periodontitis, probiotics, nanoparticles, siRNA, rheumatoid arthritis

## Abstract

**Background:**

Rheumatoid arthritis (RA) represents a primary public health challenge, which is a major source of pain, disability, and socioeconomic effects worldwide. Several factors contribute to its pathogenesis. Infections are an important concern in RA patients, which play a key role in mortality risk. Despite major advances in the clinical treatment of RA, long‐term use of disease‐modifying anti‐rheumatic drugs can cause serious adverse effects. Therefore, effective strategies for developing novel prevention and RA‐modifying therapeutic interventions are sorely needed.

**Objective:**

This review investigates the available evidence on the interplay between various bacterial infections, particularly oral infections and RA, and focuses on some potential interventions such as probiotics, photodynamic therapy, nanotechnology, and siRNA that can have therapeutic effects.

## Graphical abstract

This study aimed to investigate the available evidence on the interplay between various bacterial infections, particularly oral infections and rheumatoid arthritis and focuses on some potential interventions such as probiotics, photodynamic therapy, nanotechnology, and siRNA that can have therapeutic effects. Different bacterial pathogens alone or in inter‐bacterial interactions directly associate with the pathogenesis of RA. Potential interventions such as probiotics, photodynamic therapy, nanotechnology, and siRNA can be considered for rheumatoid arthritis treatment.

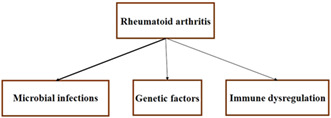

## 1 BACKGROUND

Rheumatoid arthritis (RA) is an inflammatory joint disease, with an overall prevalence of 0.46% worldwide.^1^ The disease is characterized by bone erosion, loss of articular cartilage, chronic synovial inflammation, causing joint pain, swelling, stiffness, and disability in performing physical functions.^2^, ^3^ Although all joints are subject to the disease, RA usually affects the joints of the feet, knees, and hands.^4^ The clinical manifestations of RA are usually not limited to joints but can turn into a systemic disorder involving the blood vessels, kidneys, lungs, heart, and liver. Furthermore, RA patients may suffer from nonspecific complications such as weight loss, fatigue, and malaise.^5^ The presence of either rheumatoid factor (RF) or anti‐citrullinated peptide antibodies (ACPAs) has usually been used as the hallmark of RA.^6^ This disease is multifactorial lying on genetic as well as environmental factors triggering its pathogenesis.^7^ Environmental factors such as obesity and diet, smoking, infections, and microbiota have been recognized as risk factors to develop RA in the predisposed population.^8^ HLA and some non‐HLA genes were also associated with susceptibility to RA. It was established that HLA alleles HLA‐DRB1*01, HLA‐DRB1*04, HLA‐DRB1*13, and DRB1*15 are linked to RA susceptibility.^9^

Infections have long been known as a main player in the pathogenesis of RA. Some of the pathogens can involve multiple routes of the immune system, potentially triggering several immune processes.^10^ Bacteria‐derived lipopolysaccharide (LPS) could spread into the blood and be transported to the joints. Hence, LPS could stimulate innate immune receptors in the synovium, cartilage, and bone.^11^ The development of biofilms is considered to be a driving force behind persistent infections. LPS has a significant role in biofilm formation.^12^ Moreover, endogenous and microbial ligands can activate toll‐like receptors (TLRs) and trigger an immune response in patients' derived cells. In particular, bacterial peptidoglycan and LPS induced the expression of chemokines IL‐6 and (C‐X‐C motif) ligand 8 (CXCL8), via TLR‐2 binding, in RA synovial fibroblast. ILs are a key element in infiltrating and maintaining the inflammatory cells within the synovium membrane.^13^ Furthermore, macrophages with a high‐level expression of TLR‐2 resulted in an improper response to bacterial cell wall peptidoglycan.^14^

Persuasive research has suggested a possible link between the immune response against oral infections and the development of RA through the production of enzymes by oral pathogens.^6^, ^15^, ^16^, ^17^ Pathobiology of the disease itself, immunocompromising comorbidities, lifestyle, and immunosuppressive drugs appear to play an important role in creating the conditions for the development of this correlation as well.^18^ Qiao et al. reported that patients infected by periodontal pathogens had a 69% greater risk for RA than individuals without periodontal disease.^19^

The utilization of disease‐modifying antirheumatic drugs (DMARDs) like methotrexate (MTX) is applied as first‐line therapy for RA, but long‐term use of MTX could not continue because of adverse effects and drug resistance.^20^, ^21^ To overcome the side effects, many researchers have investigated techniques to develop targeted delivery strategies. However, intra‐articular injection provides the best‐targeted therapy, frequent joint needling is usually necessary. Hence, this treatment choice might intensify the risk of infection. Also, RA involves all joints throughout the whole body. Therefore, local administration of inflamed joints is not a suitable option.^22^ The new approach for tissue‐specific delivery of drugs, as well as enhanced drug bioavailability in the inflamed synovium while reducing off‐target unwanted adverse effects, are very favorable.^23^ A detailed understanding of the pathogenesis of RA will help us to develop complementary therapies such as probiotics, photodynamic therapy (PDT), nanostructures, and siRNA against RA in the pharmaceutical market.

This review aims to achieve a comprehensive understanding of bacterial populations in the oral cavity, which may support the idea that oral infections are a main modifiable risk factor for life‐threatening diseases. Furthermore, the new therapeutic options will also be discussed how targeting RA.

## 2 RA IMMUNOPATHOGENESIS

RA is a chronic inflammatory disease involving impaired function of both the innate and adaptive immune systems.^24^ Macrophages, neutrophils, mast cells, and natural killer (NK) cells are part of the innate immune system involved in the development of the inflammatory process in the joint. Antigen‐presenting cells (APCs), such as macrophages, and dendritic cells (DCs), stimulate inflammation and release pro‐inflammatory products, such as tumor necrosis factor‐alpha (TNF‐α), cytokines interleukin‐1B, 6, 18, 23, reactive oxygen species (ROS), and matrix‐degrading enzymes involved in cartilage and bone destruction.^25^ Especially TNF‐α is produced during the inflammatory process, mainly by activated monocytes/ macrophages.^26^

Neutrophils have been known as a key contributing factor in the pathogenesis of RA. They increase inflammatory activity and tissue destruction by releasing pro‐inflammatory cytokines, enhanced oxidative stress, granules containing destructive enzymes, and release of neutrophil extracellular trap (NET) and leading to cartilage and bone destruction.^24^ Furthermore, TLRs activation plays a major role in RA pathogenesis. In RA patients, the aberrant activation of TLRs renders chronic inflammatory processes.^14^ The TLR system recognizes LPS (TLR4), peptidoglycans and lipoteichoic acid (TLR2), and unmethylated CpG DNA (TLR9) from bacteria.^27^ In addition, macrophages express TLR‐2, resulting in an improper response to the bacterial peptidoglycan.^14^ Many of these observations could be explained in RA patients, with upregulated responses to TLR‐2 and TLR‐4 ligands of blood monocytes and synovial macrophages.^28^ Additionally, type 17 helper T‐cells (Th17) have a crucial role in RA pathogenesis. IL‐17A cytokines can also mediate fibroblast‐like synoviocytes (FLS) and osteoclast activation, recruitment, and activation of neutrophils, synovial macrophages to secrete osteoclastogenic factors such as TNF‐α and IL1‐β.^29^

## 3 The ROLE OF PERIODONTAL INFECTIONS IN RA

Periodontitis as a common infectious disease has attracted much attention in the field of public health. Periodontitis leads to progressive damage such as gingival recession, alveolar bone resorption, progressive resorption of periodontal supporting tissues, and eventual tooth loss.^30^ A recent meta‐analysis found that patients with periodontitis had a 69% greater risk of RA compared to healthy groups (OR = 1.70, 95%CI: 0.75–3.85, *p* < 0.001).^19^

*Porphyromonas gingivalis* is a major periodontal pathogen that may be an important threat due to disturbing host immune homeostasis. LPS, gingipains, collagenase, proteases, fimbriae, lectins, capsule, and superoxide dismutase (SOD) are among the strategies employed by *this pathogen* to increase bacterial colonization and destroy host periodontal structures. In addition, virulence factors enhance the adhesion of *P. gingivalis* with other bacteria and biofilm build‐up. Bacterial virulence factors also modulate or interfere with inflammatory response and disrupt the host immune response to escape bacterial clearance.^31^, ^32^ *P. gingivalis* expresses the citrullinating enzyme peptidyl arginine deiminase (PAD). Citrullination, a post‐translational modification, leads to loss of tolerance to self‐proteins in genetically susceptible individuals, inducing an immune response driving RA onset. Therefore, *P. gingivalis* infection causing protein citrullination could induce the generation of ACPAs and subsequent RA development. The presence of ACPA has been related to anti‐*P. gingivalis* titres in RA patients.^33^ Interestingly, this pathogen may alter the TLR response, subvert IL‐8, or alter the complement cascade.^34^

Neutrophils as the primary effectors of the innate immune system, immobilize and kill a broad range of microbes via NET formation. NETs are networks composed of chromatin, intracellular granules, and antimicrobial peptides with high bactericidal potential that form an extracellular matrix‐like structure.^35^ Although the antimicrobial defense structures of NETs are beneficial, accumulated NETs or delayed clearance of NETs might intensify the tissue damage and represent a source of autoantigens; indeed, ACPA‐positive RA reacts strongly with histones found in NETs.^36^ The potential immunogenic role of NETs is associated with the PAD4 activity, which arises the citrullination of histones and Eno, leading to a breakdown of immune tolerance to citrullinated epitopes.^37^ Additionally, *P. gingivalis* induces NETosis in a gingipain‐dependent manner.^38^

*Prevotella intermedia*, part of the sub‐gingival microbiota, is an anaerobic Gram‐negative bacterium. *P. intermedia* plays a role in the initiation and progression of periodontitis by inducing a variety of pro‐inflammatory cytokines such as IL‐8, IL1‐β, proteases, and matrix metalloproteinases (MMPs), and macrophage inflammatory proteins.^39^ *P. intermedia* does not express the *PAD* gene but facilitates human PAD activity via the induction of activated neutrophils as the main source of citrullinated antigens.^40^ Two genes from *P. intermedia* have been characterized, *nucA* and *nucD*, encoding secreted nucleases.^41^ Nucleases can block phagocytosis by neutrophils, which could lead to increased periodontal pathogenicity and the release of endogenous PAD.^40^ In addition, this bacterium is known to induce the production of MMP‐1 and MMP‐8 in human periodontal ligament cells. These MMPs are also notably present in RA individuals.^42^, ^43^ *P. intermedia* increases the synthesis of prostaglandin E2 from arachidonic acid via inducing cyclooxygenase‐2 (COX‐2). In particular, overexpression of the COX‐2 enzyme was detected in RA.^44^

*Fusobacterium nucleatum* is a periodontal anaerobic bacterium associated with various forms of human diseases such as RA. The innate immune cells are made upby cells such as macrophages, neutrophils, mast cells, and NK cells contribute to the development of inflammatory responses in joints.^27^ The binding of NK cells to *F. nucleatum* activates a wide range of inflammatory mediators implicated in the pathogenesis of the periodontal disease.^45^ The balance between pro‐/anti‐inflammatory cytokines expression is maintained during periodontal full health. *F. nucleatum* exacerbates inflammation after the release outside the oral cavity or during dysbiosis. For example, *F. nucleatum* induces inflammatory responses through the TLR4 pathway.^45^ TLRs activation is very critical in the onset and progression of autoimmune diseases, such as RA. Patients with RA have exhibited raised levels of TLR2 and TLR4 in leukocytes.^46^ Moreover, *F. nucleatum* has a powerful stimulatory effect on inflammatory cytokines, IL‐6, IL‐8, and TNF‐α.^45^ APCs population, such as macrophages, and effector cells, increase inflammation and contribute to bone and cartilage destruction by releasing a variety of pro‐inflammatory factors.^25^ In particular, TNF‐α found crucial in the pathogenesis of the disease by increasing inflammatory cytokine levels, activating macrophages and lymphocytes.^47^

*Porphyromonas endodontalis* is an anaerobic, gram‐negative, black‐pigmented, asaccharolytic bacteria associated with periodontitis and infected root canals. *P. endodontalis* has a lower ability to bind gingival cells than *P. gingivalis* because it does not harbor the *PAD* gene.^6^, ^48^ *P. endodontalis* also does not possess a trypsin‐like enzyme associated with periodontal tissue destruction and does not display hemagglutination activity.^49^ However, *P. endodontalis* produces collagenases and protease enzymes that can also play a key role during tissue destruction.^48^ Another major virulence factor of *P. endodontalis* is LPS, which releases the secretion of inflammatory cytokines network and promotes bone destruction.^50^ *P. endodontalis* was dramatically increased in the saliva microbiota profiles of patients with early‐onset RA.^6^

*Tannerella forsythia* is an obligate anaerobe related to periodontal disease.^51^ *T. forsythia* does not contain fimbriae, and would therefore not colonize and invade periodontal tissue. However, it expresses BspA, a surface adhesion protein, in the absence of fimbriae. The BspA could utilize the *P. gingivalis* fimbrial protein‐like domains and induces the strong production of inflammatory cytokines in mammalian cells. These features provide a greater correlation between *P. endodontalis* and *T. forsythia*.^52^ Moreover, BspA has a role in a strong coaggregation with *F. nucleatum*.^53^ In cellular activation, BspA stimulates the release of bone‐resorbing pro‐inflammatory cytokines and IL‐8 from monocytes and gingival epithelial cells (GECs), respectively in a TLR‐2‐dependent manner.^51^ In addition, the lipoprotein fractions containing ester‐bound fatty acids can stimulate human gingival fibroblasts and monocytic cells to trigger pro‐inflammatory cytokines, notably IL −6 and TNF‐α.^54^ Overexpression of TNF‐α in joint synovial fluid leads to induce lymphocytes to aggregate into the inflamed joint site. Overexpressed TNF‐α stimulates the elevation of pro‐inflammatory cytokines such as IL‐1β and IL‐6, and enhances the production of collagenase and MMPs, which cause changes in subchondral bone and cartilage destruction. IL‐6 can also mediate the production of autoantibodies and RF in RA patients. In the joint, IL‐6 activates endothelial cells, resulting in the production of IL‐8, monocyte chemokine, and highly expresses adhesion molecules. Accumulation of adhesion molecules increases the leukocytes in the inflamed area. IL‐6 can also stimulate synovial cell proliferation and osteoclast activation, and finally lead to pannus formation. IL‐6 and IL‐1β, along with an increased synthesis of MMPs, are principal in articular cartilage damage.^55^ Likewise, *P. intermedia* and *T. forsythia* have been detected in synovial fluid from RA patients using the checkerboard DNA–DNA hybridization.^56^

**FIGURE 1:**
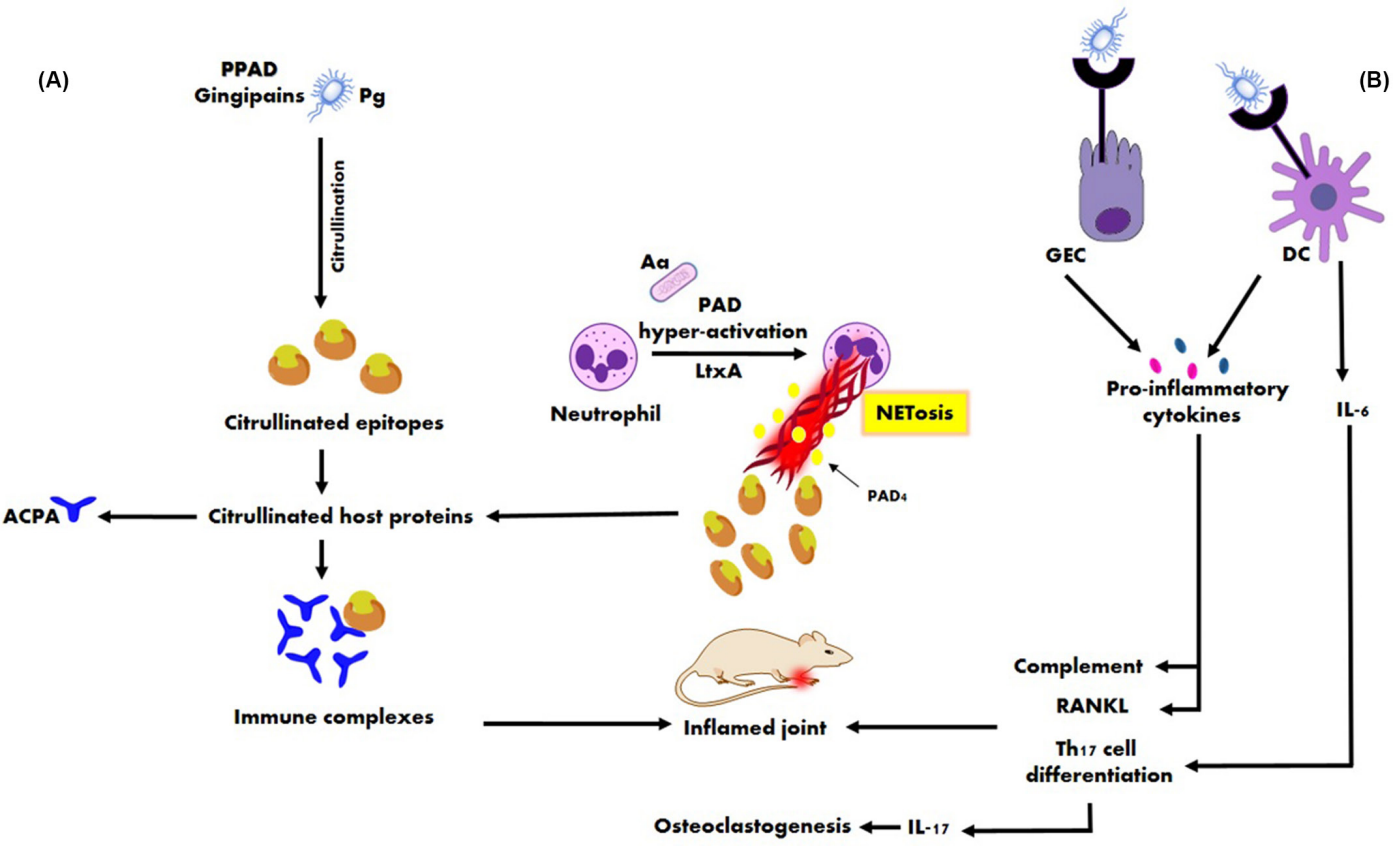
Role of periodontal bacteria in ACPAs generation and RA development. (A) *Porphyromonas gingivalis* citrullinate proteins including host proteins by the combined action of gingipains followed by citrullination by PPAD. Alternatively, the citrullinated proteins are produced through LtxA‐mediated Netosis by *Aggregatibacter actinomycetemcomitans*. Thus, both pathogens can lead to an RA‐specific ACPAs response. ACPAs: anti‐citrullinated protein antibodies, PPAD: peptidyl‐arginine deiminase, LtxA: leukotoxin A, RA: rheumatoid arthritis, APC: antigen‐presenting cell. (B) In the periodontal pockets, receptors on GECs and phagocytes, such as DCs recognize *Porphyromonas gingivalis‐associated* virulence factors. Bacteria–host cell interactions stimulate cytokines and chemokines that activate the complement pathway, RANKL, and the differentiation of T helper cells, which contribute toward joint destruction. RANKL, receptor activator for nuclear factor‐κB ligand; GECs, Gingival epithelial cells; DCs, Dendritic cells; Pg, *Porphyromonas gingivalis*; Aa, *Aggregatibacter actinomycetemcomitans*.

*Aggregatibacter actinomycetemcomitans* is a facultative anaerobic oral bacterium associated with the pathogenesis of aggressive and chronic periodontitis.^57^ This bacterium expresses several virulence factors, including LPS, adherence factors, biofilm, and toxins.^58^ Leukotoxin (LtxA) is an important protein toxin produced by *A. actinomycetemcomitans* and plays a critical role in the subversion of the host immune response.^58^ *A. actinomycetemcomitans* induces hypercitrullination in human neutrophils through the activity of LtxA, the main target of autoantibodies in RA.^59^ Furthermore, LtxA induces the production of IL‐1β and IL‐18 cytokine by macrophages. These cytokines are the principal mediators in the immunopathogenesis of RA.^60^ As well, LtxA promotes NET release via a CD11/CD18 (LFA‐1) route. LFA‐1, a cell surface receptor for LtxA on neutrophils, is involved in a downstream signaling cascade that may also participate in ROS generation. The increased production of ROSs is necessary for NET release.^61^ The increased ROS following NETosis can activate the receptor activator of the nuclear factor kappa‐B ligand (RANKL) expression on osteoblast and stimulate osteoclast activation.^62^ The role of periodontal bacteria in ACPAs generation and RA development is depicted in Figure 1A, B.

*Peptostreptococcus micros* is an anaerobic Gram‐positive bacteria predominated in periodontitis and infected dental root canals.^63^ Yoshioka, et al. report that LPS released from *A. actinomycetemcomitans* can attach to *P. micros*, resulting in a population of bacteria that could be a potent stimulant for human macrophages. *P. micros* might help cytokine induction in a periodontal lesion, thus favoring inflammation.^64^ *P. micros* can adhere to host epithelial cells and interact with periodontopathogenic bacteria, including *F. nucleatum* and *P. gingivalis*.^65^ *P. micros* cell wall induced the secretion of a high level of IL‐6 cytokine by macrophages. IL‐6 enhances bone resorption.^63^

*Treponema denticola*, an anaerobic oral spirochete, could be the main contributor to periodontal disease.^66^ *T. denticola* Eno is a virulence factor, with higher degrees of homology with the human Eno. It is also served as a plasminogen‐binding receptor, which assists in inflammatory cell invasion.^67^ Interaction between anti‐ Eno antibodies and Eno‐positive cells that express in synovial fluid and peripheral blood mononuclear cells may contribute to the elevated TNFα serum levels in patients with RA, and the circulating TNFα may contribute to the progression of periodontal disease.^68^, ^69^ In addition, *P. gingivalis*, *T. forsythia*, *F. nucleatum*, and *A. actinomycetemcomitans* contain human Eno‐homologous proteins.^70^

**FIGURE 2:**
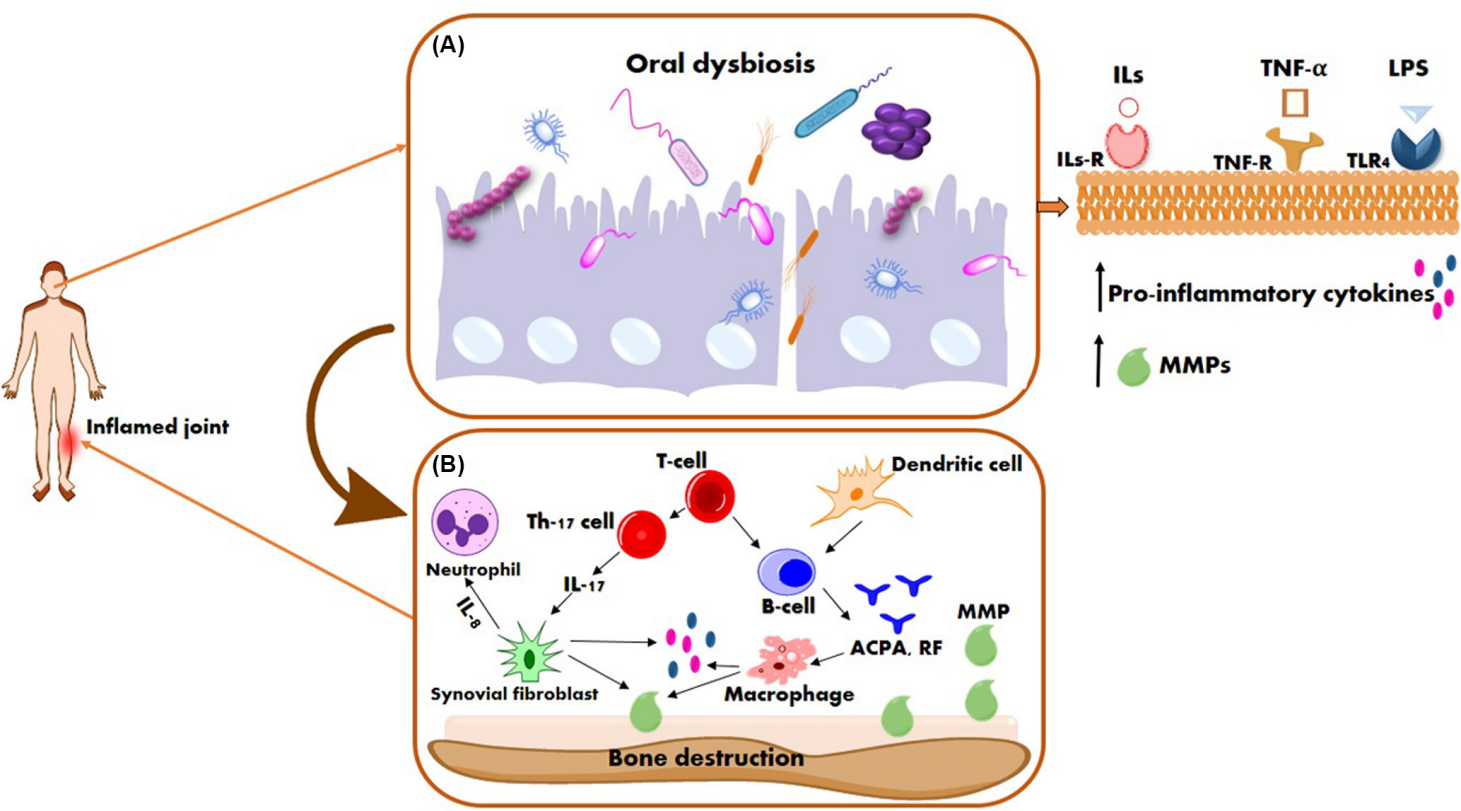
Schematic illustration of the links between rheumatoid arthritis and periodontal Infections. (A) An oral dysbiosis onto the tooth surface initiates innate immunity by producing pro‐inflammatory mediators in response to bacterial LPS (via the toll‐like receptor). (B) Immune cells will produce pro‐inflammatory mediators such as ILs, TNF, and MMPs, which also contribute to the augmentation of the immune response. IL‐17 induces the production of pro‐inflammatory cytokines and MMPs. The interactions among B and T cells and APCs play a major role in immune response activation, leading to the production of autoantibodies and the development of bone resorption. The autoantibodies contributed to the inflammatory process and resulted in bone and cartilage damage. LPS, lipopolysaccharide; ILs, interleukins; TNF, tumor necrosis factor; MMPs, matrix metalloproteinases; APCs, antigen‐presenting cells.

*Filifactor alocis* is a Gram‐positive anaerobic bacterium and has been detected in endodontic infections, and periodontal disease.^71^ It interacts with other bacteria, specifically *P. gingivalis*. This interaction increases invasive capability and biofilm formation. *F. alocis* expresses various virulence factors and can promote the expression of pro‐inflammatory factors and proteases.^72^ *F. alocis* can convert the arginine to ornithine via an enzymatic pathway without the citrulline production step.^73^ The L‐ornithine‐converting enzyme was increased approximately 1.5‐fold in early‐onset RA patients' microbiota, supporting a potential association between *F. alocis* and RA progression.^6^ The link between oral dysbiosis with the pathogenesis of RA is depicted in Figure 2.

## 4 THERAPEUTIC INTERVENTIONS

Although drug molecules, predominantly glucocorticoids, and DMARDs, are unchanged as first‐line drug treatment; novel approaches are appealing options to address this challenge.^74^ The most common type of adverse effect of DMARDs is an elevated risk of bacterial, fungal, and viral infections.^75^ Glucocorticoids accelerated bone resorption by increasing osteoclast precursors.^76^ The development of new therapies and drugs for RA has emphasized on inducing the FLS to stop proliferation. This proliferation is related to the activation of specific intracellular signaling pathways. Such inflammation, if not treated at a particular point in time, leads to vascular invasion and cartilage damage by erosion, which causes limited mobility and joint pain. Synovectomy, a standard treatment against RA, is an invasive procedure, destructive, and requires prolonged rehabilitation periods. In recent years, minimally invasive treatments have gained increased attention.^77^

**FIGURE 3:**
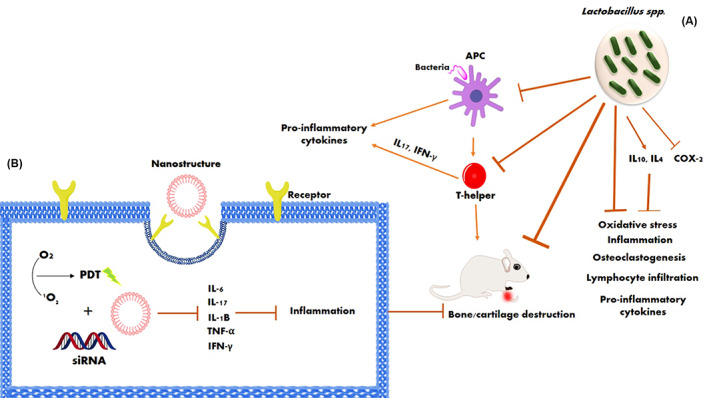
Schematic illustration of the role of (A) *Lactobacillus* spp. and (B) nanostructures in rheumatoid arthritis therapy. Photodynamic therapy/siRNA complex nanostructures prevent the expression of pro‐inflammatory cytokines and inhibit bone and cartilage destruction.

Bacteria can also be used as carriers for drug delivery systems.^78^ Maddaloni et al. fabricated *Lactococcus lactis* as a delivery system for IL‐35, a unique anti‐inflammatory cytokine. IL‐35 plays a substantial role in the induction and development of T and B regulatory cells.^79^ Various studies have shown treatment intervention with *Lactobacillus* spp. reduce arthritic scores and pro‐inflammatory cytokines like IL‐17, IL‐1, IL‐6, and TNF‐α, and induce anti‐inflammatory cytokines such as IL‐4 and IL‐10 by inhibiting the COX‐2 enzyme. They decrease oxidative stress, suppressed *Th17* cell‐mediated secretion of pro‐inflammatory cytokines, diminish swelling, cartilage damage, and lymphocyte infiltration in joints, and release short‐chain fatty acids.^44^, ^80^, ^81^ A mixture of *lactobacillus* probiotics (*L. rhamnosus* GR‐ 1 and *L. reuteri* RC‐14) showed substantially lower levels of IL‐1α, IL‐6, IL‐12p70, and TNF‐α.^82^ So et al. have shown that the oral administration of *L. casei* displays protective effects against RA in rodents. This micro‐organism induces IL‐10 whilst the expression of pro‐inflammatory cytokines such as IL‐1, IL‐2, IL‐6, IL‐12, IL‐17, interferon‐gamma, COX‐2, and TNF‐α remains restricted (Figure 3A).^83^

The use of complementary therapies such as PDT could improve the sense of well‐being and increase the likelihood of remission.^77^ PDT involves a photosensitizing agent, which is excited by visible light at specific wavelengths in the presence of oxygen. Next, the excitation energy gives rise to ROS.^84^ Therefore, PDT based on the photosensitizing agents induces cell death in inflammatory cells participating in the joint. Hence, combining PDT with standard treatments can improve the control of bone and cartilage damage in the treated joint.^77^ Numerous studies reported that PDT significantly reduced pro‐inflammatory cytokine levels such as IL‐6, IL‐17, and TNF‐α.^13^, ^85^, ^86^

Nanotechnology can be a promising therapeutic strategy for RA. Compared with drugs, nanoparticles (NPs) are more s in circulation and have a longer half‐life, so they are widely applied as carriers for drug delivery. Moreover, the NPs demonstrated good sustained‐release function. They enhance the retention time of drugs in the joint cavity.^87^ Chitosan is a natural cationic polysaccharide molecule with similar biological and chemical attributes to components of the bone extracellular matrix.^88^ Chitosan NP containing zinc gluconate resulted in reduced expression of pro‐inflammatory cytokines and enhanced levels of SOD expression.^89^ Sun et al. formed a self‐assembled structure with entrapped Cu/Zn‐SOD. These polymeric NPs demonstrated good anti‐inflammatory and antioxidant activities in a rat adjuvant‐induced arthritis model (Figure 3B).^90^

Macrophages are dynamic cells that play a pivotal role during the pathogenesis of RA. The activated RA synovial macrophages express the folate receptor β (FR‐β). Triptolide (TP) is a molecule with anti‐inflammatory features, but its clinical application to treat RA has been limited due to many disadvantages like poor solubility, low bioavailability, and extremely high toxicity. Liposomes (Lips) are biocompatible spherical vesicles which used as preferred carriers for the delivery of therapeutic agents.^91^ The main benefits of Lips include biocompatibility, high stability, high drug loaded to carrier ratios, nontoxicity, and protective features of drugs.^92^ Folate‐modified TP‐Lips (FA‐TP‐Lips) target activated macrophages, thereby, improving the safety and effectiveness of treatment in RA. FA‐TP‐Lips with anti‐inflammatory activities inhibited osteoclastogenesis without causing cytotoxic effects. Furthermore, Lips could prolong the circulation lifetime of TP in vivo, as well as show significant anti‐inflammatory and cartilage protective effects with lower toxicity compared with the TP group alone, thereby providing a promising new treatment for RA.^93^ Lips as drug delivery systems have helped through the modulation of inflammation and cytokine secretion. A study designed nano‐drug delivery systems for loading avocado soy unsaponifiable (ASU), which synergistically enhanced ASU anti‐inflammatory effects with Lip.^94^, ^95^ The ASU combination inhibits nuclear factor kappa B (NF‐kB), monocyte migration as well as decreases the side effects due to the co‐delivery of ASU within Lips vesicles.^96^, ^97^ The loaded Lip with ASU has shown positive effects in controlling and reducing joint inflammation in osteoarthritis models. ASU/liposome combination may be effective in controlling RA joint degradations. However, further investigation is needed on RA models.

In addition, NPs can be used to overcome drawbacks and improve the potency and effectiveness of therapeutic agents.^98^, ^99^ The copper sulfides (Cu_7.2_S_4_) NPs plus near‐infrared (NIR) laser irradiation (808 nm, 1 W/cm^2^) have been applied in PDT against RA. While Cu‐based nanomaterials can serve as photosensitizer agents. The combination of Cu_7.2_S_4_ NP with NIR light showed an increase in ROS production. Meanwhile, this combination reduces the level of pro‐inflammatory proteins.^100^ Zhao et al. showed PDT using tetra sulphonatophenyl porphyrin‐titanium dioxide nanowhiskers have a strong therapeutic effect in RA.^13^

Small interfering RNA (siRNA) showed great potential to decrease main inflammatory cytokines such as TNF‐α and IL‐1β.^21^ A siRNA does not easily deliver to cells through passive diffusion due to its high molecular weight, hydrophilicity, and negative charge. Of course, due to susceptibility to endonuclease cleavage, and short half‐life in serum, an efficient delivery system is essential for the clinical application of siRNA.^101^ Duan et al. designed the folate‐conjugated PEGylated Lip that could load the siRNA/ calcium phosphate NPs (siRNA/CaP) in its core while MTX will be loaded in the lipid shell of the Lips. An effective combination therapy using NPs inhibited the NF‐kB signaling pathways and reduced the production of pro‐inflammatory cytokines, and also avoid the MTX side effects.^21^ In RA, the inflammatory signals induced through TNF‐α and IL‐1β degrade the inhibitory kB protein, resulting in the release of NF‐kB and transfer to the nucleus where they activate multiple inflammatory signals and RA progression.^102^ TNF‐α siRNA has revealed evidence of the beneficial therapeutic effects in animal models of RA. Aldayel et al. developed a novel acid‐sensitive sheddable PEGylated solid‐lipid NP comprised of TNF‐α siRNA, lecithin, and cholesterol. This formulation shows a high encapsulation efficiency and a low burst siRNA release and can target inflamed sites after intravenous administration in mouse models that do not respond to MTX. The complexes significantly reduced RA‐induced bone loss, paw thickness, and histopathological scores.^103^ Likewise, Lee et al. fabricated anti‐TNF‐α with polymerized siRNA/ thiolated glycol chitosan NP for the treatment of RA. This compound remarkably inhibited inflammation and bone erosion in an animal model.^104^

## 5 CONCLUSIONS

The presented evidence indicates that different bacterial pathogens alone or in inter‐bacterial interactions directly associate with the pathogenesis of RA. The link between microbial dysbiosis and RA should open up interesting therapeutic prospects at a time when we are in urgent need of new alternative treatments. Eventually, clinical trials involving complementary therapies are necessary to provide a definite answer as to the applicability of each approach. In addition, basic and comprehensive researches at the molecular level are needed to fully understand the mechanism of the mentioned therapeutic interventions. Meanwhile, combination therapy should be investigated in detail to increase the retention time and efficiency of therapeutics. Moreover, formulation‐related safety concerns and obstacles should be evaluated systematically.

## AUTHOR CONTRIBUTIONS

Literature search, collection, interpretation, and original draft writing; S.A.; Conceptualization, design, and construction of the conceptual framework of the review; S. A, N.C., A.P., R.G.; Review and editing of the manuscript; S.A., A.P. All authors have read and agreed to the submitted final version of the manuscript.

## FUNDING INFORMATION

This work was funded by Tehran University of Medical Sciences of Medical Science and Health Services (No. 1401‐4‐209‐64414).

## CONFLICT OF INTEREST STATEMENT

The authors declare that they have no competing interests.

## CONSENT FOR PUBLICATION

All of the authors approved the manuscript for publication.

## Data Availability

All data are available from the corresponding author.
